# Rapidly growing proliferative pulmonary chondroma: A case report

**DOI:** 10.1016/j.ijscr.2022.107776

**Published:** 2022-11-13

**Authors:** Tsuyoshi Uchida, Hirochika Matsubara, Mamoru Muto, Aya Sugimura, Yuichiro Onuki, Hiroyuki Nakajima

**Affiliations:** Department of Thoracic Surgery, Yamanashi University, Shimokato 1110, Chuo, Yamanashi, Japan

**Keywords:** Pulmonary chondroma, Carney's triad, Lobectomy, Case report

## Abstract

**Introduction:**

Pulmonary chondroma, a component of Carney's triad, is commonly unilateral and multiple, and progresses slowly. Herein, we report a case of a chondrogenic tumour that grew and proliferated during follow-up.

**Presentation of case:**

A female patient in her 20s presenting with a cough was found to have a 1.4-cm nodule in the left lung on computed tomography (CT). After 18 months' follow-up, CT revealed that the original nodule had increased to 2.2 cm, and a new 1.3-cm nodule had appeared. She was then referred to our hospital and underwent a robot-assisted lower lobectomy of the left lung. The tumour was diagnosed as a chondrogenic tumour. She had no problems after the surgery or during follow-up; other signs of the Carney's triad were ruled out. Twenty-six months postoperatively, there was no evidence of recurrence.

**Discussion:**

One report suggests that the growth of pulmonary chondroma is slow, but the present case showed an increase in both the size and number of tumours within 2 years without any symptoms. The chondroma did not recur after the surgery, though her pulmonary tumours had grown and proliferated rapidly. Furthermore, it has been reported that an average of 8.4 years is needed for another sign of Carney's triad to appear; therefore, careful follow-up should be continued.

**Conclusion:**

This report suggests that pulmonary chondroma can grow and proliferate rapidly and asymptomatically, and can be controlled by complete resection.

## Highlights

• Pulmonary chondroma can grow and proliferate rapidly and asymptomatically.
• Pulmonary chondroma can be controlled by complete resection.
• Pulmonary chondroma is a subset of the Carney's triad.
• It has been reported to take an average of 8.4 years for the second sign of the Carney's triad to appear.

## 1 Introduction

Pulmonary chondroma, one of the components of Carney's triad, is commonly unilateral and multiple and progresses slowly. It is difficult to diagnose as a benign tumour without a biopsy when the tumour increases and proliferates. We herein report a case of a chondrogenic tumour that increased and proliferated during regular follow-up. This case report has been reported in line with the SCARE Criteria [1].

## 2 Presentation of case

**Fig. 1.**
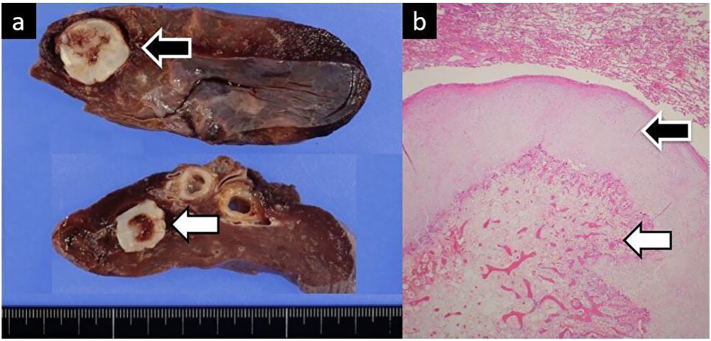
Computed tomography (CT) at initial presentation. a) CT shows a 1.4-cm nodule in the lower lobe of the left lung (black arrow). b) Positron emission tomography (PET) shows very little accumulation (white arrow).

**Fig. 2.**
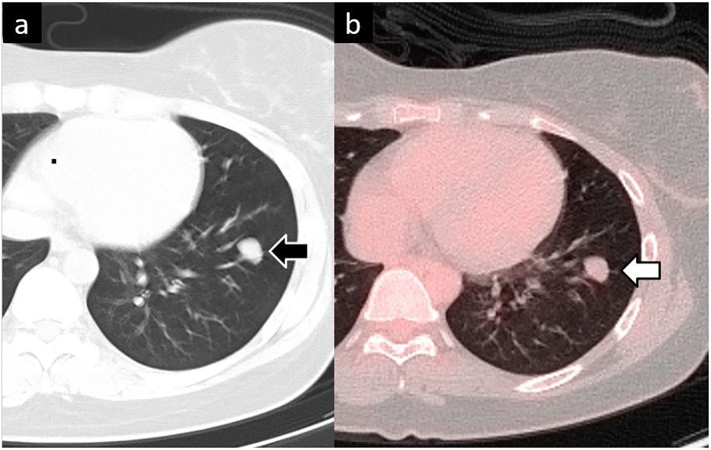
a) A chest radiograph showing the nodule noted on CT. The black arrow indicates the tumour. b) Chest radiograph of the nodule after 18 months of follow-up. The white arrow indicates the tumour.

**Fig. 3.**
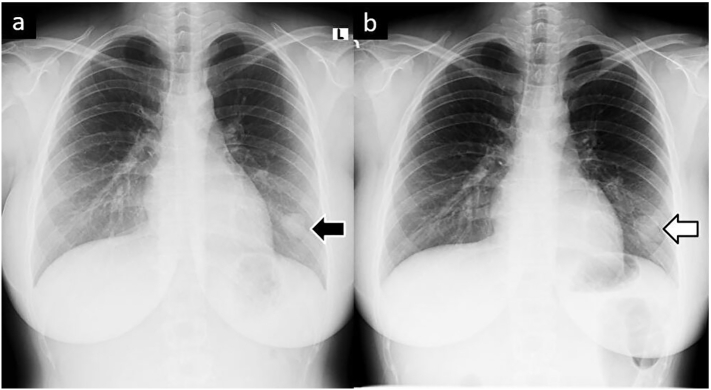
First CT after being referred to our hospital. The black arrows indicate that the original nodule had increased to 2.2 cm, and a new 1.3 cm nodule had appeared. The primary nodule had a chondrocyte component (white arrow), but the new daughter nodule did not (white triangle).

**Fig. 4.**
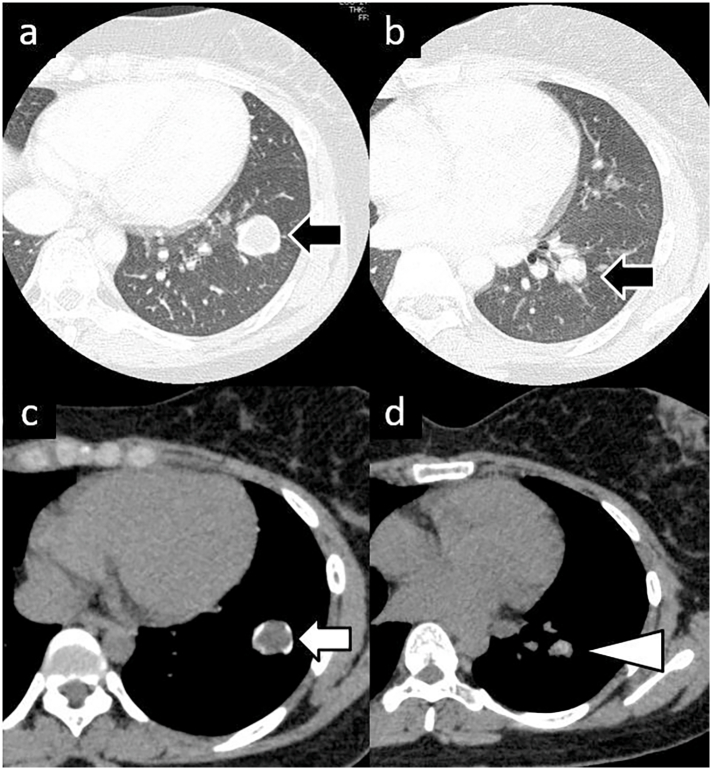
a) Gross findings. Two white, smooth-surfaced nodules were observed (white and black arrows). b) Microscopic findings (hematoxylin and eosin staining). Bone and fatty tissue (white arrow) surrounded by a cartilage matrix (black arrow) were observed and a diagnosis of pulmonary chondroma was made.

A woman in her 20s presented to the emergency department of the care facility, where she has been followed-up for 4 years, chiefly complaining of an asthma-like cough. *A*1.4-cm nodule was observed in the lower lobe of her left lung on computed tomography (CT) (Fig. 1a). Subsequently, positron emission tomography (PET) showed a very small accumulation (Fig. 1b). At this point, it was assumed to be a benign tumour, and the patient and her parents refused surgical resection. Therefore, she was followed up with a chest radiography every 3 months. At the sixth follow-up visit, the chest radiograph indicated slight enlargement of the nodule, along with a second, smaller nodule which newly had appeared (Fig. 2). CT revealed that the original nodule had increased in size to 2.2 cm, while the new nodule measured 1.3 cm (Fig. 3a, b). The patient was then referred to our hospital. Her general condition was good and tumour markers (carcinoembryonic antigen, cytokeratin 19 fragment, squamous cell carcinoma antigen) were not significantly elevated. Due to the rapid growth and proliferation of the tumour and the absence of the chondrocyte component in the new daughter nodule (Fig. 3c,d), malignant disease could not be ruled out and we decided to perform a surgical resection. The patient had no significant medical history but had a Brinkman Index of over 100, despite being in her early 20s. She had no other specific physical problems and underwent a robot-assisted lower lobectomy of the left lung. The frozen tumour section was intraoperatively diagnosed as a chondrogenic tumour; therefore, mediastinal lymph node dissection was not performed. Histological examination of the resected specimen showed the features of chondroma: bone and fatty tissue surrounded by a cartilage matrix (Fig. 4). The patient had no postoperative complications and was followed up after other signs of Carney's triad were ruled out. Twenty-six months after surgery, there was no evidence of recurrence.

## 3 Discussion

This case suggests that pulmonary chondroma can grow and proliferate rapidly and asymptomatically. The tumour was discovered by an emergency CT scan taken when the patient presented chiefly complaining of cough. Although previous reports have reported cough as a clinical manifestation of pulmonary chondroma [2], her cough was only coincidental and was not associated with tumour growth or multiplication. Although one report suggests that the growth of pulmonary chondroma is slow [3], our report showed an increase in both the size and number of the tumours within 2 years. There have been reports of chondrosarcoma with a large mass and lymph node metastasis 2 months after the onset of cough and chest pain [4]; however, there was no malignant transformation in this case, although the tumour growth was relatively rapid.

This case also suggests that pulmonary chondroma can be controlled by complete resection. Hiroto et al. reported a case with multiple bilateral granular shadows, but there was no specific mention of therapeutic intervention for the remaining granular shadows other than those which were excised and biopsied [5]. Helmy et al. reported the results of a biopsy of bilateral nodules and finding of GIST 21 years later; therefore, a molecular targeted drug (imatinib) was administered for 2 years [6]. The present case involves a nodule that grew and multiplied over 2 years without recurrence in the patient's lung or other signs of Carney's triad, such as GIST or adrenal paraganglioma, 26 months after lower lobectomy.

Pulmonary chondroma is a subset of Carney's triad and it has been reported to take an average of 8.4 years for the second sign to appear [7]; therefore, careful follow-up should continue.

## Patient's perspective

I was told it was probably a benign tumour, so I was very surprised when I found out that it had grown and increased in size over the last 2 years. I was scared of the surgery, but I am glad they did it with a small scar. I was told that I might develop other symptoms, but so far there are no signs of them. I have regular check-ups and I feel safe.

## Consent

Written informed consent was obtained from the patient for publication of this case report and accompanying images. A copy of the written consent is available for review by the Editor-in-Chief of this journal on request.

## Funding

This research did not receive any specific grants from funding agencies in the public, commercial, or not-for-profit sectors.

## Provenance and peer review

Not commissioned, externally peer-reviewed.

## Ethic approval

The review board of Yamanashi University provided an ethics exemption for this study.

## Guarantor

Tsuyoshi Uchida.

## CRediT authorship contribution statement

Tsuyoshi Uchida: Writing-original draft, Data curation, Formal analysis.

Hirochika Matsubara: Writing – review & editing.

Mamoru Muto: Writing – review & editing, Data curation, Formal analysis.

Aya Sugimura: Writing – review & editing, Data curation, Formal analysis.

Yuichiro Onuki: Writing – review & editing.

Hiroyuki Nakajima: Writing – review & editing, Supervision.

## Declaration of competing interest

The authors have no competing interests to declare.
